# Apigenin inhibits fibrous scar formation after acute spinal cord injury through TGFβ/SMADs signaling pathway

**DOI:** 10.1111/cns.13929

**Published:** 2022-07-30

**Authors:** Zhengxin Jin, Lige Tian, Ying Zhang, Xiaodi Zhang, Jianning Kang, Hui Dong, Nana Huang, Liuzhu Pan, Bin Ning

**Affiliations:** ^1^ Central Hospital Affiliated to Shandong First Medical University Shandong First Medical University & Shandong Academy of Medical Sciences Jinan China; ^2^ Tianjin Medical University Tianjin China; ^3^ School of Clinical Medicine Weifang Medical University Weifang China; ^4^ Jinan Central Hospital, Cheeloo College of Medicine Shandong University Jinan China

**Keywords:** apigenin, fibrous scar, spinal cord injury, TGFβ/SMADs

## Abstract

**Aim:**

To investigate the effect of apigenin on fibrous scar formation after mouse spinal cord injury (SCI).

**Methods:**

The pneumatic impactor strike method was used to establish an SCI model. Mice were intraperitoneally injected with 5 mg/kg or 20 mg/kg apigenin daily for 28 days after SCI. The Basso Mouse Scale (BMS) score, hematoxylin–eosin staining, and immunohistochemical staining were used to assess the effect of apigenin on scar formation and motor function recovery. Western blotting and qRT‐PCR were used to detect the expression of fibrosis‐related parameters in spinal cord tissue homogenates. NIH‐3 T3 cells and mouse primary spinal cord fibroblasts, α‐Smooth muscle actin (α‐SMA), collagen 1, and fibronectin were used to evaluate apigenin's effect in vitro. Western blotting and immunofluorescence techniques were used to study the effect of apigenin on TGFβ/SMADs signaling.

**Results:**

Apigenin inhibited fibrous scar formation in the mouse spinal cord and promoted the recovery of motor function. It reduced the expression of fibroblast‐related parameters and increased the content of nerve growth factor in vivo, decreasing myofibroblast activation and collagen fiber formation by inhibiting TGFβ‐induced SMAD2/3 phosphorylation and nuclear translocation in vitro.

**Conclusion:**

Apigenin inhibits fibrous scar formation after SCI by decreasing fibrosis‐related factor expression through TGFβ/SMADs signaling.

## Graphical abstract

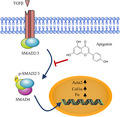

## 1 INTRODUCTION

Spinal cord injury (SCI) is a devastating injury that brings sensory and motor dysfunction to patients, causing serious social and economic impacts.^1^ Studies have shown that the recovery of nerve and motor function is closely related to the formation of scars and regeneration of nerves after SCI.^2^, ^3^ Scars formed after SCI are classified as fibrous and glial scars.^4^ Fibrous scars are formed by the activation of fibroblasts around the dura mater and blood vessels in the injured area, invading the hyperplastic hypertrophy of the injured area. Fibrous scars inhibit axonal regeneration and functional recovery, resulting in permanent functional deficits. ^5^

Transforming growth factor β (TGFβ) is one of the main catalysts that activate resting fibroblasts and is a multifunctional cytokine‐mediating immune regulation, angiogenesis, and the regulation of fibrotic activity. In the TGFβ/SMADs signaling pathway, SMADs proteins act as direct substrate proteins for TGFβR action, participating in and regulating its cellular signaling. There are nine types of SMAD proteins, of which receptor‐regulated SMAD2 and 3, co‐regulated SMAD4, and inhibitory SMAD7 are mainly involved in TGFβ signaling.^6^ Combined with our earlier findings, the TGFβ/SMADs signaling pathway was significantly activated, with TGFβ/SMADs pathway‐related protein mRNA levels being significantly increased.^7^ Therefore, the TGFβ/SMADs signaling pathway necessarily is a key factor after SCI (Appendix S1).

Apigenin is a flavonoid widely found in nature and extracted mainly from celery, parsley, thyme, chamomile, and onion.^8^ Studies have indicated that it plays an important role in antioxidation,^9^ and has antiinflammation,^10^ antitumor (prostate,^11^ ovarian,^12^ breast,^13^ lung,^14^ gastric,^15^ etc.), and antifibrosis roles, among others. Wang et al. demonstrated that apigenin attenuates TGFβ‐stimulated cardiac fibroblast differentiation and extracellular matrix production by targeting the miR‐155‐5p/c‐Ski/Smad pathway.^16^ Hicks et al. confirmed apigenin is a potential antifibrotic agent targeting hepatic stellate cells.^17^ However, its role in SCI has not been reported yet.

This article aimed to investigate the role and specific molecular mechanism of apigenin in regulating fibroblasts after SCI. We hypothesized that apigenin may attenuate fibrous scar formation after SCI by inhibiting TGFβ/SMADs signaling.

## 2 METHODS

### 2.1 Chemicals

TGFβ was from Peprotech. Apigenin (>99% purity) was from MCE (MedChemExpress). SIS3 was from MCE (MedChemExpress).

### 2.2 Cell culture

The mouse embryonic fibroblast cell line NIH‐3 T3 was from the National Collection of Authenticated Cell Cultures, and mouse primary spinal cord fibroblasts were from Procell. NIH‐3 T3 cells were cultured in Dulbecco's modified Eagle's medium (Gibco) with 10% newborn calf serum (Gibco), 100 U/ml penicillin–streptomycin, glutamax (Invitrogen), and a sodium pyruvate 100 mM solution (Gibco). The mouse primary spinal cord fibroblasts were placed in complete culture medium (CM‐M162, Procell). All cells were maintained at 37°C in a humidified atmosphere with 5% CO_2_.

### 2.3 Cell counting kit‐8 (CCK‐8) assay

NIH‐3 T3 cells in the log phase were trypsinized and resuspended in complete medium. Then, 100 μl of cell suspension (about 7 × 10^3^ cells) was added to each well of a 96‐well culture plate. After culturing for 24 and 48 h in different concentrations of apigenin, 10 μl of CCK‐8 reagent (AmyJet) were added to each well, followed by incubation for 2 h. Subsequently, a spectrophotometer (Thermo) was used to detect the absorbance at 450 nm.

### 2.4 Animals

C57BL/6 mice (6–8 weeks old, 18–22 g body weight) were purchased from Jinan Pengyue Laboratory Animal Breeding Co., Ltd. Mice were housed under standard conditions (12 h light/dark cycles with the lights on from 07:00 to 19:00), and food and water provided ad libitum. The relevant animal procedures were approved by the Jinan Central Hospital (No. JNCH‐202114). C57BL/6 mice were randomly divided into four groups: Sham (T8–T10 laminectomy only), control (T8–T10 laminectomy and injury), apigenin 5 mg/kg (T8–T10 laminectomy and injury, apigenin 5 mg/kg), apigenin 20 mg/kg (T8–T10 laminectomy and injury, apigenin 20 mg/kg).

### 2.5 SCI model establishment

Mice were injected intraperitoneally with 3% pentobarbital (30 mg/kg; Sigma‐Aldrich), and a spinal cord impactor (RWD, 68100) was used after T8–T10 laminectomy to expose the spinal cord at a speed of 1 m/s and a depth of 2 mm. The success of SCI modeling was judged by significant visualization of the injury site, transient spasms in the hind limbs and tail, and a postoperative loss of motor and sensory function. The surgical area was sutured layer by layer. The bladder was manually squeezed three times a day until spontaneous micturition resumed.

### 2.6 BMS score assessment

Normal mice were placed in an open area 1 day before surgery to familiarize them with the environment. Each mouse was scored for BMS on postoperative days 1, 3, 7, 10, 14, 18, 21, 24, and 28, with an observation period of 5 min for each score. The experiment was performed by an experimenter familiar with the scoring rules and scored immediately after observation.

### 2.7 qRT‐PCR

Total RNA was extracted with a kit (AG21017, AG11728; Accurate Biotechnology [Hunan] Co., Lte.) and reverse transcribed. A SYBR Green Premix Pro Taq HS qPCR kit (AG11701; Accurate Biotechnology [Hunan] Co., Lte.) was used qRT‐PCR, performed in a Light Cycler 480 II (Roche) real‐time polymerase chain reaction system. The mRNA levels of the target gene were calculated by the 2^−ΔΔCT^ method and normalized to the mRNA levels of the glyceraldehyde‐3‐phosphate dehydrogenase (GAPDH) gene to determine fold changes in the relative expression of the target gene. The sequences of forward and reverse primers used are shown in Table 1.

**TABLE 1:** The primer sequence of gene

Gene	Primer sequence, 5′–3′	
	Forward	Reverse
GAPDH	TGTCTCCTGCGACTTCAACA	GGTGGTCCAGGGTTTCTTACT
Col1α1	GTGAGACAGGCGAACAAG	CCAGGAGAACCAGGAGAA
Col1α2	CTCCTGGCAATCGTGGTTCA	GCCAACATTTCCAGGAGACC
Col4α1	AGGAACGACTACTCTTACTG	CACTGCGGAATCTGAATG
Actα2	TGAAGAGCATCCGACACT	GCCTGAATAGCCACATACAT
Fn	CCATTCCACCTTACAACAC	CAAGCCAGACACAACAAT
Ngf	TGCCAAGGACGCAGCTTTC	TGAAGTTTAGTCCAGTGGGCTTCAG

### 2.8 Western blotting

Cells or tissues were collected and lysed to prepare protein samples, which were separated by SDS‐PAGE and transferred to nitrocellulose (NC) membranes. NC membranes were blocked with 5% skimmed milk in Tween 20 (TBST) tris‐buffered saline for 1.5 h at room temperature and shaken overnight at 4°C with primary antibody. Subsequently, the NC membrane was incubated with the corresponding secondary antibody for 1 h at room temperature. Finally, chemiluminescence reagents (Cat. No: BL520B, Biosharp) were added to the NC membrane, which was developed in a gel imager (FluorChem M, Proteinsimple). Signals were analyzed by Image J software (National Institutes of Health).

The primary antibodies were anti‐SMAD2/3 (1:1000, Cell Signaling Technology), anti‐Phospho‐SMAD2/3 (1:1000, Cell Signaling Technology), anti‐α‐SMA(1:1000, Proteintech), anti‐Phospho‐AKT (1:1000, Cell Signaling Technology), anti‐AKT (1:1000, Cell Signaling Technology), anti‐Phospho‐p44/42 MAPK (ERK1/2) (1:1000, Cell Signaling Technology), anti‐p44/42 MAPK (ERK1/2) (1:1000, Cell Signaling Technology), anti‐SMAD4 (1:1000, Proteintech), anti‐SMAD7 (1:1000, Santa Cruz), anti‐FN (1:1000, Proteintech), and anti‐GAPDH, (1:5000, Proteintech).

### 2.9 Hematoxylin and eosin staining (H&E staining)

Spinal cord tissues 5 mm above and below the injury site were removed and fixed in 4% paraformaldehyde (Solarbio) for 24 h, followed by dehydration and paraffin embedding. Serial 5‐μm sections were cut with a microtome (Leica [China]). Sections were deparaffinized stepwise with xylene and different concentrations of ethanol. Hematoxylin staining was performed for 5 min for water and differentiation solution washing. Sections were sequentially dehydrated in 85% and 95% graded alcohol for 5 min and stained with eosin for 5 min. Sections were dehydrated and mounted in neutral gum. Representative images were observed and captured using an electron microscope (Olympus).

### 2.10 Immunofluorescence staining and immunohistochemical staining

Tissue sections were analyzed as per the kit (sp‐9000, ZSGB‐BIO) manufacturer's instructions, using the antibodies α‐SMA (1:200, Proteintech) and CSPG (1:100, SIGMA‐ALDRICH).

### 2.11 Statistical analysis

All data are expressed as the mean ± SD. BMS scores were analyzed by two‐way analysis of variance with Bonferroni post hoc correction, and multiple group comparisons were analyzed using one‐way analysis of variance (anova) with Bonferroni post hoc correction. GraphPad Prism v8.0 software was used for statistical analysis. *p* value <0.05 was considered statistically significant.

## 3 RESULTS

### 3.1 Effects of different concentrations of apigenin on NIH‐3 T3 cells

**FIGURE 1:**
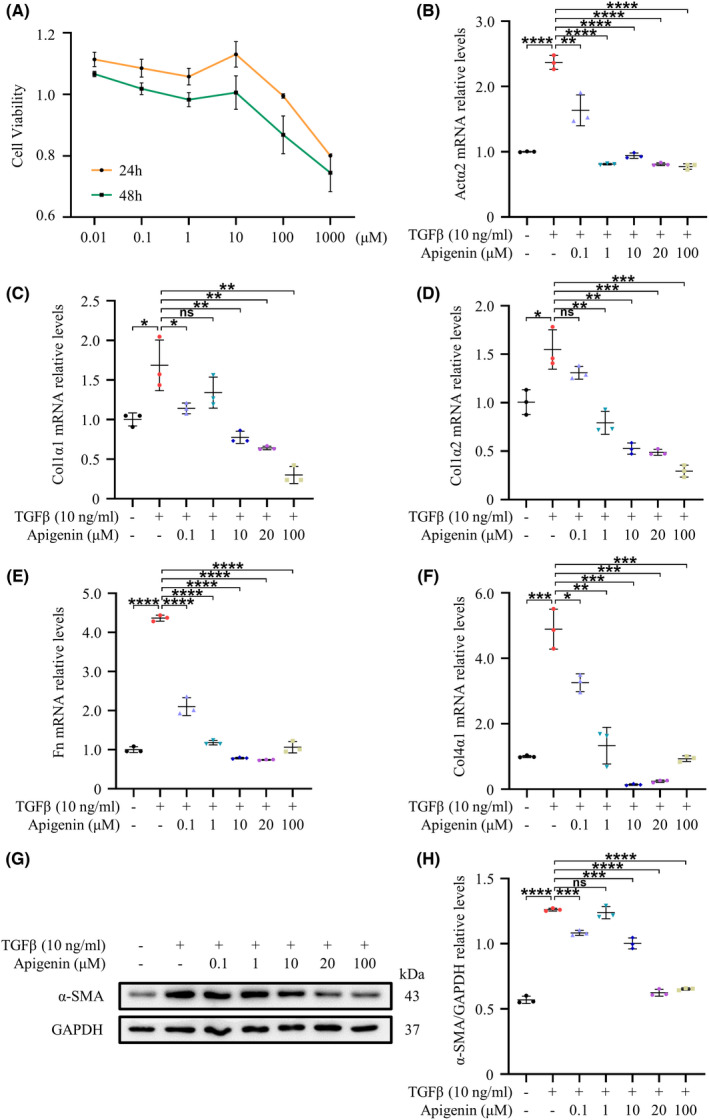
Effects of different apigenin concentrations on NIH‐3 T3 cells. (A) CCK‐8 analysis of the viability of NIH‐3 T3 cells. (B–F) qRT‐PCR was used to detect the expression of *Actα2*, *Col1α1*, *Col1α2*, *Fn* and *Col4α1* in NIH‐3 T3 cells treated with different apigenin concentrations for 24 h. (G) Western blotting was used to detect the expression of α‐SMA in NIH‐3 T3 cells treated with different concentrations of apigenin for 48 h. (H) Quantification of the expression of α‐SMA. (* means *p* < 0.05; ** means *p* < 0.01; *** means *p* < 0.001; **** means *p* < 0.0001, *p* values were calculated with one‐way anova, followed by Bonferroni's multiple comparison test. *n* = 3)

To study the effects of different concentrations of apigenin on fibroblasts, we selected seven concentration gradients with a 10‐fold increase from 0.01 to 1000 μM. As shown in Figure 1A, CCK‐8 detection found that apigenin had almost no toxic effects on NIH‐3 T3. The expression of the fibrosis‐related genes *Actα2*, *Fn*, *Col1α1*, *Col1α2* and *Col4α1* in NIH‐3 T3 was increased by 1.5–4.5 times after 24 h of stimulation with 10 ng/ml TGFβ. However, all relevant parameters decreased after treatment with different apigenin concentrations (Figure 1B–F). Additionally, the expression of α‐SMA, a marker of myofibroblasts, was significantly increased after TGFβ stimulation, whereas apigenin suppressed this change (Figure 1G,H). Combining the results of qRT‐PCR and Western blotting, we finally selected the apigenin concentrations of 10 μM and 20 μM for further experiments.

### 3.2 Apigenin inhibits fibrosis expression induced by TGFβ in NIH‐3 T3 cells

**FIGURE 2:**
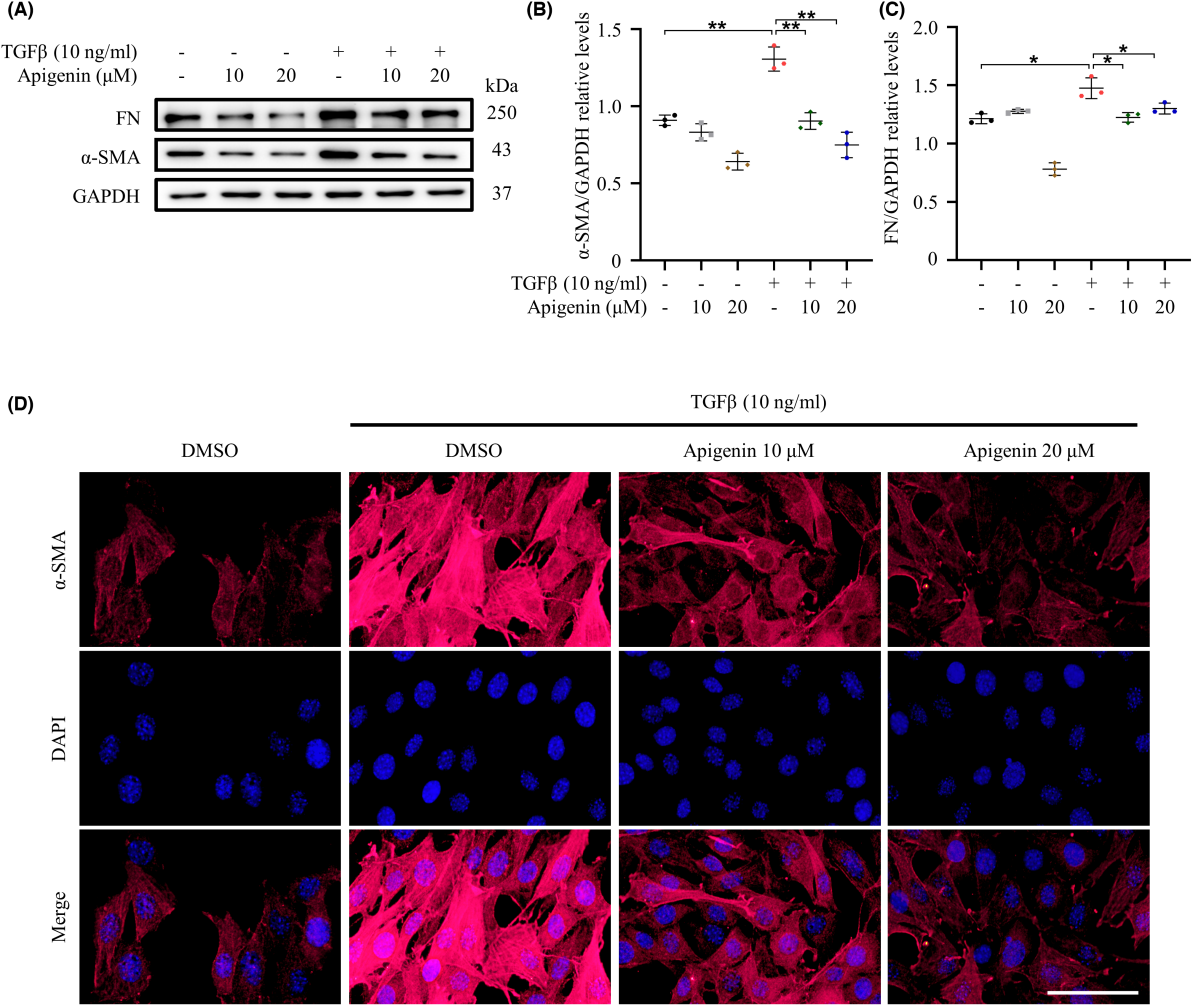
Apigenin inhibits fibrosis expression induced by TGFβ in NIH‐3 T3 cells. (A) The expression of Fn and α‐SMA in NIH‐3 T3 cells incubated with DMSO or apigenin for 24 h in the presence or absence of TGFβ was detected by Western blotting and (B, C) quantitative analysis. (D)The expression of α‐SMA, with or without TGFβ stimulation and incubation with DMSO or apigenin for 24 h, was detected by immunofluorescence (Scale bar = 12.5 μm). (* means *p* < 0.05; ** means *p* < 0.01, *p* values were calculated with one‐way anova, followed by Bonferroni's multiple comparison test. *n* = 3)

To further validate the inhibitory effect of apigenin on fibrosis, we first examined the expression of α‐SMA and fibronectin by Western blotting. The expressions of α‐SMA and fibronectin were up‐regulated after TGFβ stimulation, while they were down‐regulated after the addition of 10 μM and 20 μM apigenin (Figure 2A–C). We then examined α‐SMA expression by immunofluorescence. Cells were activated, and the expression of α‐SMA was significantly increased after TGFβ treatment. Apigenin significantly decreased the expression of α‐SMA in a concentration‐dependent manner (Figure 2D). We could conclude that apigenin can inhibit the activation of NIH‐3 T3 cells.

### 3.3 Apigenin inhibits the activation of primary mouse spinal cord fibroblasts

**FIGURE 3:**
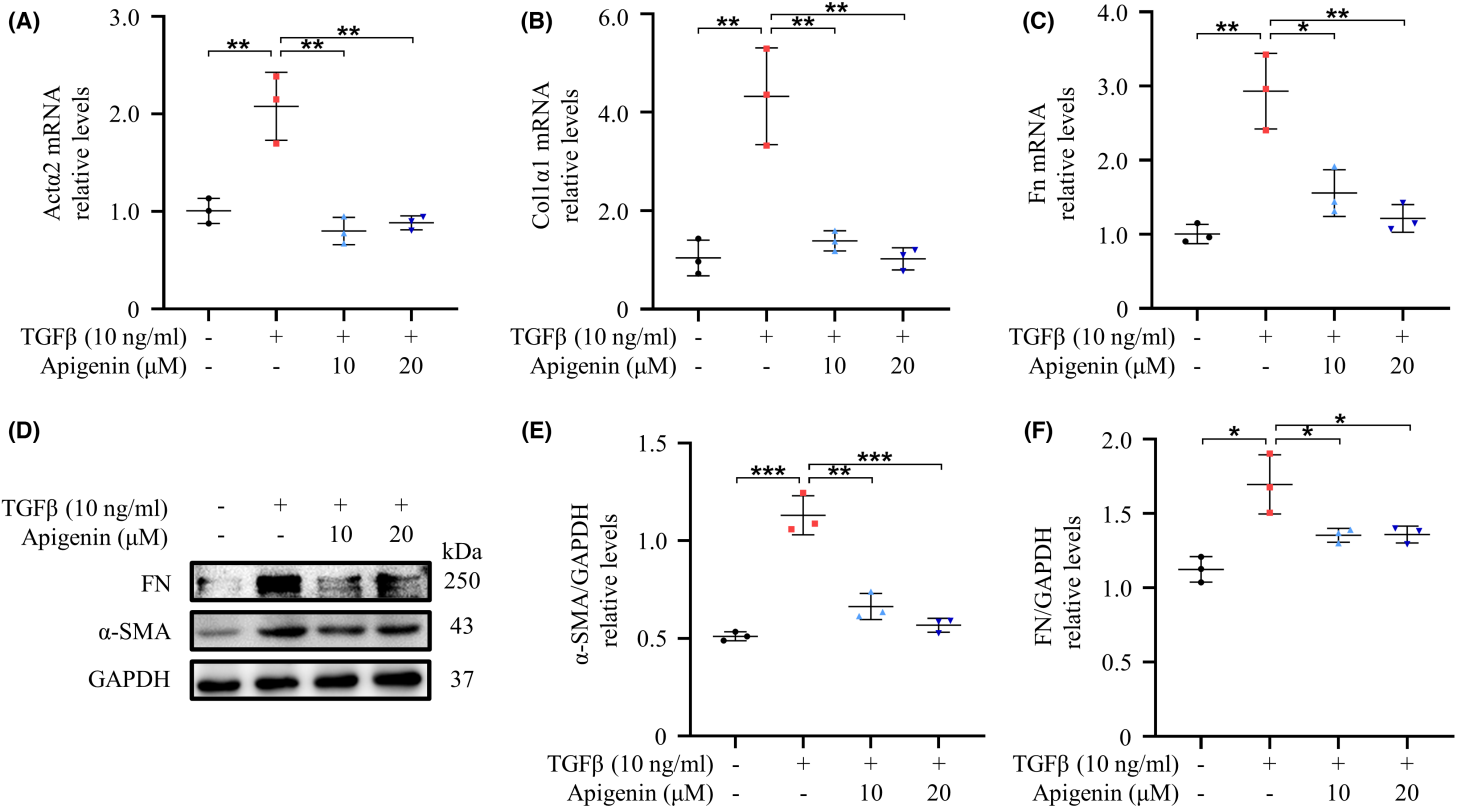
Apigenin inhibits the activation of primary mouse spinal cord fibroblasts. After primary mouse spinal cord fibroblasts were stimulated by TGFβ and incubated with DMSO or apigenin, fibrosis‐related mRNA and protein expression were detected by (A–C) qRT‐PCR and (D) Western blotting. Quantitative analysis of α‐SMA and Fn protein expression is shown in (E, F). (* means *p* < 0.05; ** means *p* < 0.01; *** means *p* < 0.001, *p* values were calculated with one‐way anova, followed by Bonferroni's multiple comparison test. *n* = 3)

Treatment with or without apigenin in primary mouse spinal cord fibroblasts indicated that apigenin could better inhibit TGFβ‐induced fibrosis‐related parameter elevation with increasing concentrations (Figure 3A–C). The protein expression of FN and α‐SMA was detected by Western blotting, and apigenin was found to be able to inhibit the TGFβ‐induced up‐regulation of α‐SMA and FN (Figure 3D–F). Our experimental results showed that apigenin significantly inhibited mRNA and protein expression in mouse spinal cord fibroblasts.

### 3.4 Apigenin promotes recovery and alleviates scar formation after spinal cord injury in mice

**FIGURE 4:**
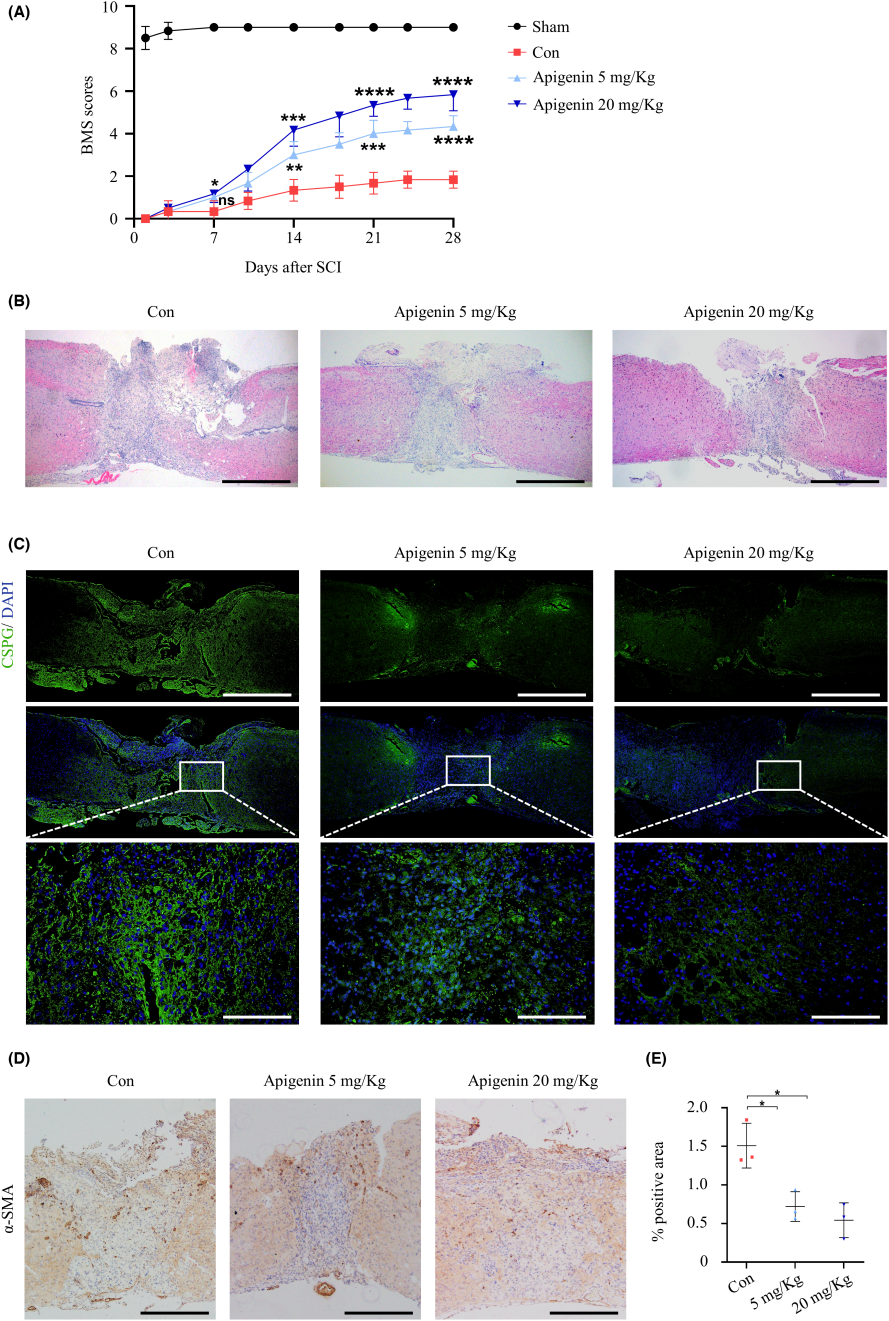
Apigenin promotes recovery and alleviates scar formation after spinal cord injury in mice. (A) BMS scores indicate the motor functional index over 28 days after SCI (*n* = 6). (B) Sections of injured spinal cord were stained with H&E (Scale bar = 50 μm). (C) The expression of CSPG was detected by tissue immunofluorescence (upper Scale bar = 50 μm, lower Scale bar = 25 μm). (D) α‐SMA was detected by tissue immunochemistry (Scale bar = 50 μm), and the analysis of positive areas is shown in (E). (ns means *p* > 0.05; * means *p* < 0.05; ** means *p* < 0.01; *** means *p* < 0.001; **** means *p* < 0.0001, *p* values of (A) were analyzed by two‐way analysis of variance, and *p* values of (E) were calculated with one‐way anova, both *p* valuesfollowed by Bonferroni's multiple comparison test. *n* = 3)

To verify the effect of apigenin on SCI, we established a mouse model of SCI using a percussion and scored BMS to evaluate the recovery of mouse motor function. The results of the BMS score showed that the motor function of mice in the apigenin group was significantly improved, and the improvement in the high concentration group was more significant than that in the low concentration group (Figure 4A). H&E staining showed that scar formation was observed at the site of tissue injury, which was reduced in the apigenin treatment group (Figure 4B). Immunofluorescence analysis of tissue sections showed that the expression of CSPG was decreased in the apigenin‐treated group compared to the control group (Figure 4C). Similarly, immunohistochemistry showed that apigenin reduced the elevation of α‐SMA after SCI (Figure 4D,E). These results demonstrated that apigenin could reduce the formation of fibrous scars after SCI in mice.

**FIGURE 5:**
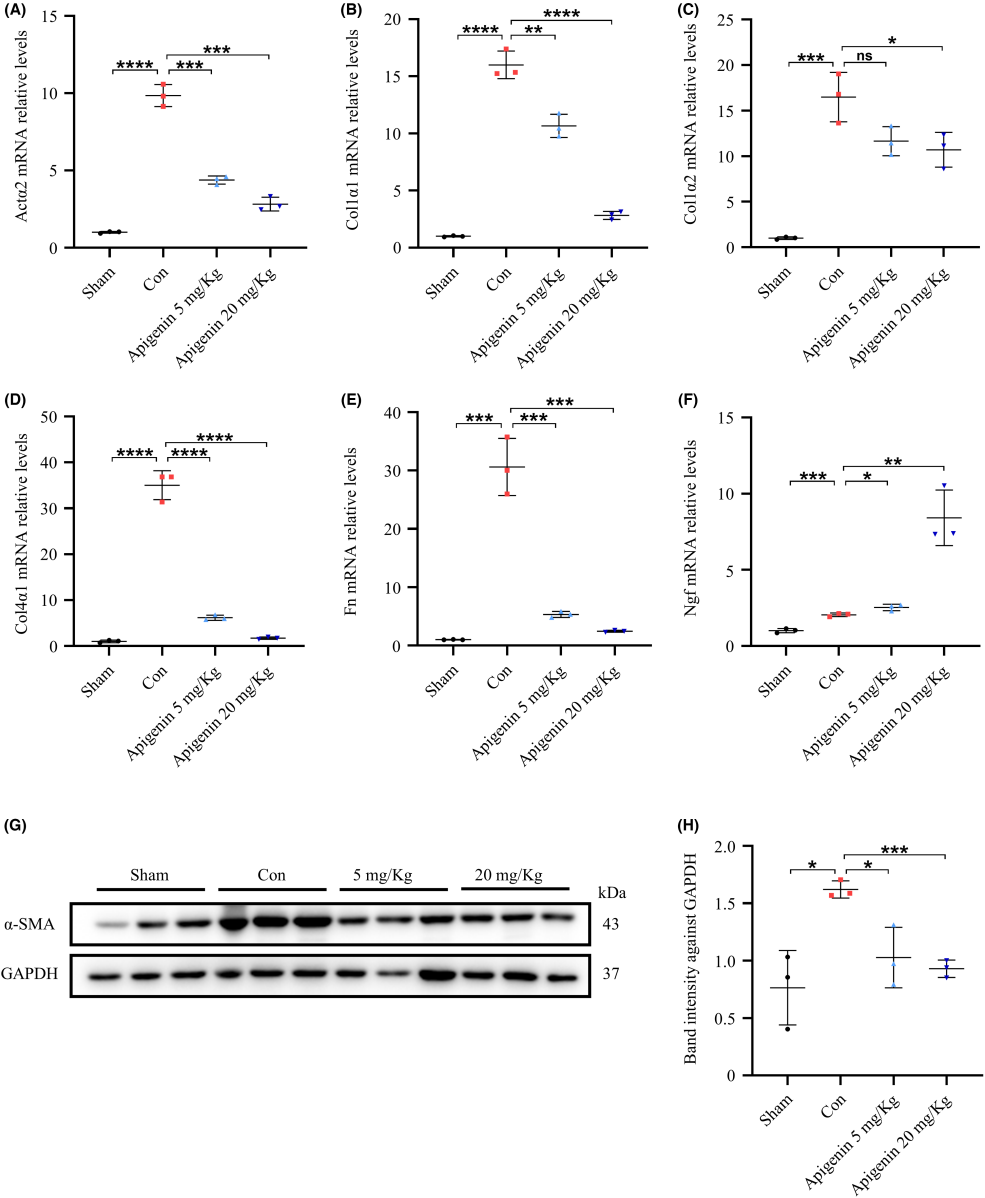
Apigenin inhibits fibrous scar formation after spinal cord injury. (A–F) Expression of *Actα2*, *Col1α1*, *Col1α2*, *Col4α1*, *Fn* and *Ngf* in spinal cord homogenates after injured 28 days detected by qRT‐PCR. (G) Protein was extracted from spinal cord tissues 28 days after spinal cord injury, and the expression of α‐SMA was detected by Western blotting. (H) Quantitative analysis of α‐SMA expression. (* means *p* < 0.05; ** means *p* < 0.01; *** means *p* < 0.001; **** means *p* < 0.0001, *p* values were calculated with one‐way anova, followed by Bonferroni's multiple comparison test. *n* = 3)

Compared with the normal group, the expression of fibrosis‐related genes in the spinal cord tissue was significantly increased by 8–33‐fold in the control group. However, the expression of *Actα2*, *Col1α1*, *Col1α2*, *Col4α1*, and *Fn* decreased significantly in a dose‐dependent manner in the apigenin group (Figure 5A–E). Interestingly, the expression of nerve growth factor (*Ngf*) in the apigenin treatment group was significantly higher than that in the control group (Figure 5F), indicating that apigenin may promote nerve regeneration by increasing the expression of *Ngf* after SCI. Spinal cord tissue proteins were extracted and the expression of α‐SMA protein was detected by Western blotting. The expression of α‐SMA decreased in the apigenin treatment group, especially in the high‐concentration treatment group (Figure 5G,H). Taken together, these results confirm that apigenin reduces scar formation after SCI in mice and promotes the expression of *Ngf* as well as the recovery of motor function.

### 3.5 Apigenin inhibits TGFβ/SMADs pathway activity

**FIGURE 6:**
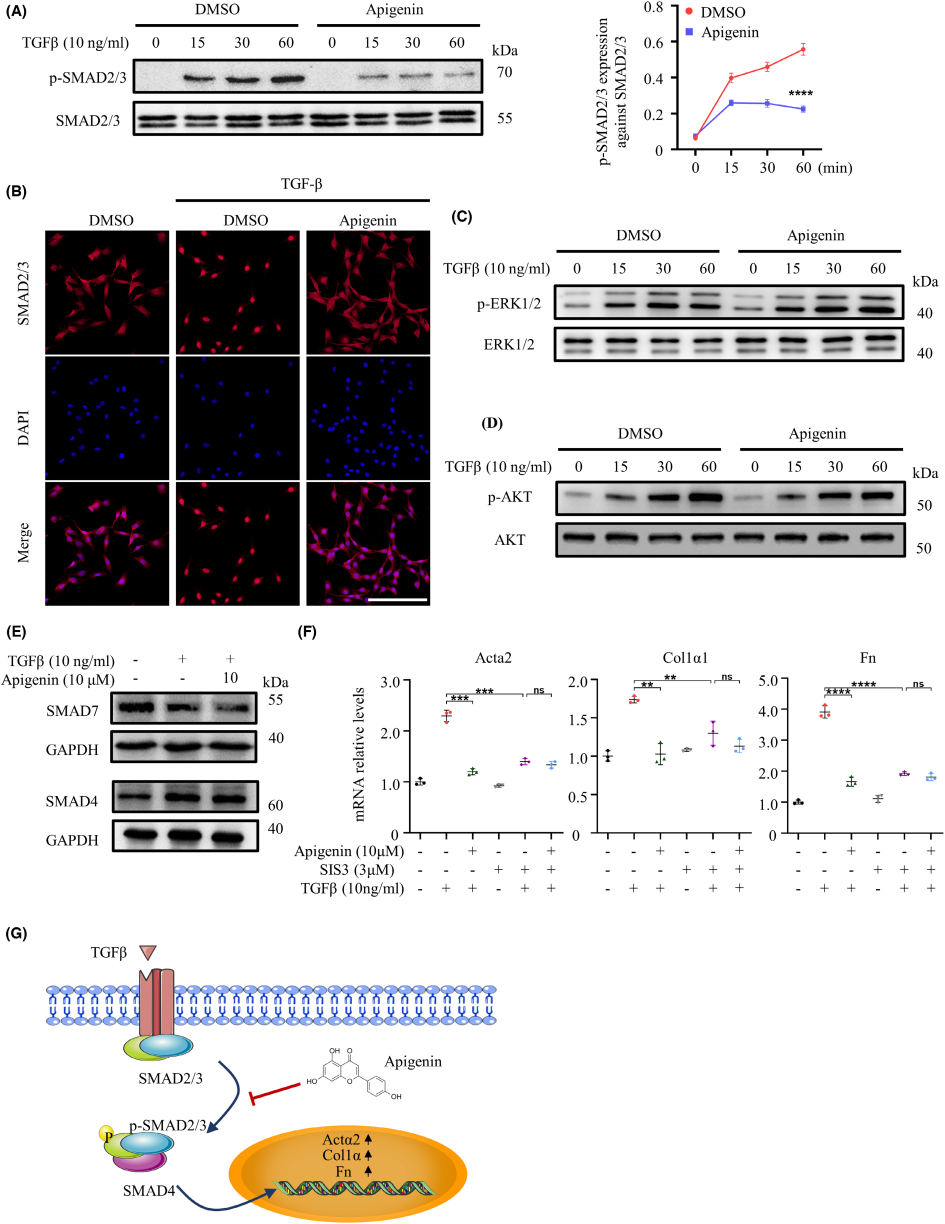
Apigenin inhibits TGFβ/SMADs signaling. (A) Expression of phosphorylated‐SMAD2/3 and SMAD2/3 proteins detected by Western blotting and quantitative analysis. (B) Expression of SMAD2/3 in NIH‐3 T3 cells detected by immunofluorescence (Scale bar = 50 μm). (C) Expression of phosphorylated ERK1/2 and ERK1/2 proteins detected by Western blotting. (D) Expression of phosphorylated AKT and AKT proteins detected by Western blotting. (E) Expression of SMAD4 and SMAD7 proteins detected by Western blotting. (F) qRT‐PCR was used to detect the expression of *Actα2*, *Col1α1*, and *Fn* in NIH‐3 T3 cells treated with apigenin or SIS3, with or without TGFβ for 24 h. (G) Schematic representation of apigenin‐alleviating fibrous scar formation after spinal cord injury by inhibiting TGFβ/SMADs signaling. (ns means *p* > 0.05; ** means *p* < 0.01; *** means *p* < 0.001; **** means *p* < 0.0001, *p* values were calculated with one‐way anova, followed by Bonferroni's multiple comparison test. *n* = 3)

The TGFβ/SMADs pathway is involved in most types of fibrosis disease.^18^ To further explore the mechanism of apigenin‐mediated inhibition of fibrous scar formation after SCI, NIH‐3 T3 cells were treated with DMSO or apigenin for 24 h. TGFβ was added with an incubation time of 0, 15, 30, or 60 min, and cellular protein was extracted to detect the expression of phosphorylated SMAD2/3 protein. The phosphorylation level of SMAD2/3 decreased continuously from 15 to 60 min of treatment (Figure 6A), which demonstrated that apigenin could inhibit the phosphorylation of SMAD2/3. Immunofluorescence staining was used to detect the localization of SMAD2/3, and the number of SMAD2/3 nuclei decreased in the apigenin‐treated group (Figure 6B). We also explored the effects of apigenin on other pathways involved in fibrosis. The stimulation of TGFβ similarly promoted the phosphorylation of ERK1/2 and AKT, while apigenin had no effect on this change (Figure 6C,D). SMAD4 and SMAD7 can regulate the activity of SMAD2/3. Apigenin did not affect the expression of SMAD4 and SMAD7 at the protein level after TGFβ stimulation for 24 h (Figure 6E). Following functional experiments in NIH‐3 T3 cells, qRT‐PCR results showed that SIS3, an inhibitor of SMAD3, was equally effective in reducing TGFβ‐induced high expression of fibrosis‐related genes at the mRNA level. However, co‐stimulation of SIS3 and apigenin did not show a better therapeutic effect compared to apigenin alone (Figure 6F). All these results show that apigenin can inhibit the phosphorylation of SMAD2/3 and its entry into the nucleus, thus inhibiting TGFβ/SMADs signaling and reducing fibrous scar formation after SCI (Figure 6H).

## 4 DISCUSSION

SCI remains a serious and difficult‐to‐treat disease. There are 15–20 cases of traumatic SCI per million people every year, and SCI causes 7 billion US dollars in losses to society every year.^19^ Patients with acute SCI usually present a permanent disability, accompanied by a number of complications such as urinary incontinence, pressure sores, and ventilatory dysfunction.^20^ Current treatment methods are mainly limited to surgical decompression and acupuncture support measures,^21^ which cannot solve the problem from the source. To find a better treatment strategy, we ought to understand the pathophysiological mechanism of and cytobiological differences after SCI.

The pathological manifestations of SCI can be divided into two injury stages. In the first stage, when the patient is injured, the severity of mechanical violence on the spinal cord directly determines SCI severity. The first stage mainly includes local edema, ischemia, hemorrhage, necrosis, and tissue laceration caused by trauma. The second stage is chronic long‐term progressive damage after injury, including mitochondrial dysfunction, local ischemia, moderate excitability, neuroinflammatory reaction, apoptosis, and scar formation.^22^

The key to neuronal regeneration after SCI lies in the recovery of the number of nerve cells and functional repair in the injured site. The disturbance of neuronal cell regeneration is mainly related to scar formation and the weakened regeneration ability of mature neuronal cells. Scars formed after SCI can be classified as fibrous and glial scars. Glial scars are traditionally believed to play a major inhibitory role in the process of axonal regeneration. Studies have shown that glial scars play a positive role in neuroprotection in the acute phase after SCI, while fibrous scars form a mechanical barrier and inhibit axonal regeneration after SCI.^23^ Fibrous scars are formed by the activation, proliferation, and migration of vascular peripheral cells and fibroblasts to the lesion center. Fibroblasts have been shown to reduce the length of neurons in vitro ^24^, ^25^ and in vivo.^26^, ^27^ Fibroblasts can secrete extracellular matrix components (such as fibronectin, type IV collagen, and laminin) and proliferate and hypertrophy, forming a mechanical barrier that hinders axonal growth recovery. Fibroblasts can also secrete axon growth inhibitory molecules (CSPGs, NG2 proteoglycan, tendon protein C, etc.) which severely hinder axonal regeneration and functional recovery. Therefore, inhibiting the activation of fibroblasts to inhibit the formation of fibrous scars is a key to functional recovery after SCI.

In recent years, small molecular drugs have received increasing attention because of their accessibility and low cost. Some articles have studied the potential of new small molecular drugs in the treatment of SCI. Some articles have even proposed a model of drug screening for SCI treatment.^28^

Apigenin is a compound found in nature and can be extracted from celery, parsley, thyme, chamomile, and onion. Studies have shown that apigenin has the effects of antiinflammation, antioxidation, weight loss, antitumor, antivirus, antifibrosis, etc. It has been reported to have a protective effect in liver, pulmonary, and myocardial fibroses. However, its role in SCI has not been reported.

In this study, the mouse embryonic fibroblast cell line NIH‐3 T3 was used to explore the effects of different concentrations of apigenin, with two concentrations selected for further study. These concentrations were used to treat the mouse embryonic fibroblast line NIH‐3 T3 and the primary mouse spinal cord fibroblast stimulated by TGFβ. Western blotting, qRT‐PCR, and immunofluorescence allowed the detection of several key fibrosis indexes (*α‐SMA*, *Fn*, *Col1α1*, *Col1α*2, and *Col4α* expression) to verify the inhibitory effect of apigenin on fibroblast activation. Next, we build a model of SCI mice, and intraperitoneally injected them with two different doses of apigenin every day. The recovery of motor function was observed by BMS score. On the 28th day, the mice were killed, and the spinal cord tissue was taken out. Tissue immunofluorescence, Western blotting, and qRT‐PCR were used to compare the formation of fibrous scars among groups, demonstrating that apigenin can inhibit the formation of fibrous scars in vivo.

The TGFβ family includes a series of structural and functional polypeptide growth factor subfamilies such as TGFβ and activin A.^29^ TGFβ family ligands can form ligand‐receptor complexes with their corresponding type I and type II receptors on the cell membrane. Type II receptors act on type I receptors, phosphorylating them and activating their kinase activity. After type I receptor activation, they quickly recruit and continue to activate downstream SMAD2/3 proteins to form SMAD2/3/4 complexes, which enter and accumulate in the nucleus to mediate transcriptional regulation.^30^ TGFβ is one of the main catalysts for activating resting fibroblasts. TGFβ signaling pathway can activate fibroblasts near the dura mater and blood vessels in the area of SCI, making fibroblasts proliferate, migrate, and secrete ECM and promoting the process of tissue fibrosis. In order to explore the mechanism of apigenin‐mediated inhibition of fibrous scar formation, we used Western blotting to detect the degree of SMAD2/3 phosphorylation after apigenin treatment with TGFβ at different times. We detected the entry of SMAD2/3 protein into the nucleus by immunofluorescence, which demonstrated that apigenin could inhibit the TGFβ/SMADs pathway.

## 5 CONCLUSION

Apigenin inhibits fibrous scar formation after SCI by decreasing the expression of fibrosis‐related factors through the TGFβ/SMADs signaling pathway.

## AUTHOR CONTRIBUTIONS

JZ helped with cell culture, animal experiments, Western blotting and real‐time polymerase chain reaction experiments; analyzed the data; and wrote the manuscript. TL participated in the study design and manuscript revision. KJ helped with cell culture and animal modeling and analyzed the data. DH helped with immunofluorescence and immunohistochemistry experiments and analyzed the data. ZY and HN helped with H&E staining experiments. ZX helped with real‐time polymerase chain reaction. NB helped with the study, analyzed the data, and wrote the manuscript. All authors approved the final version of the manuscript.

## CONFLICT OF INTEREST

The authors declare that the research was conducted in the absence of any commercial or financial relationships that could be construed as a potential conflict of interest.

## Supporting information


Appendix S1

## Data Availability

The raw data supporting the conclusions of this article will be made available by the authors. Further inquiries can be directed to the corresponding author/s.
