# Supplementary material for: Apigenin inhibits fibrous scar formation after acute spinal cord injury through TGFβ/SMADs signaling pathway
Source: CNS Neurosci Ther. 2022 Jul 30;28(11):1883–94. doi: 10.1111/cns.13929 (PMC9532920; doi:10.1111/cns.13929)

Full unedited blot for Figure 1

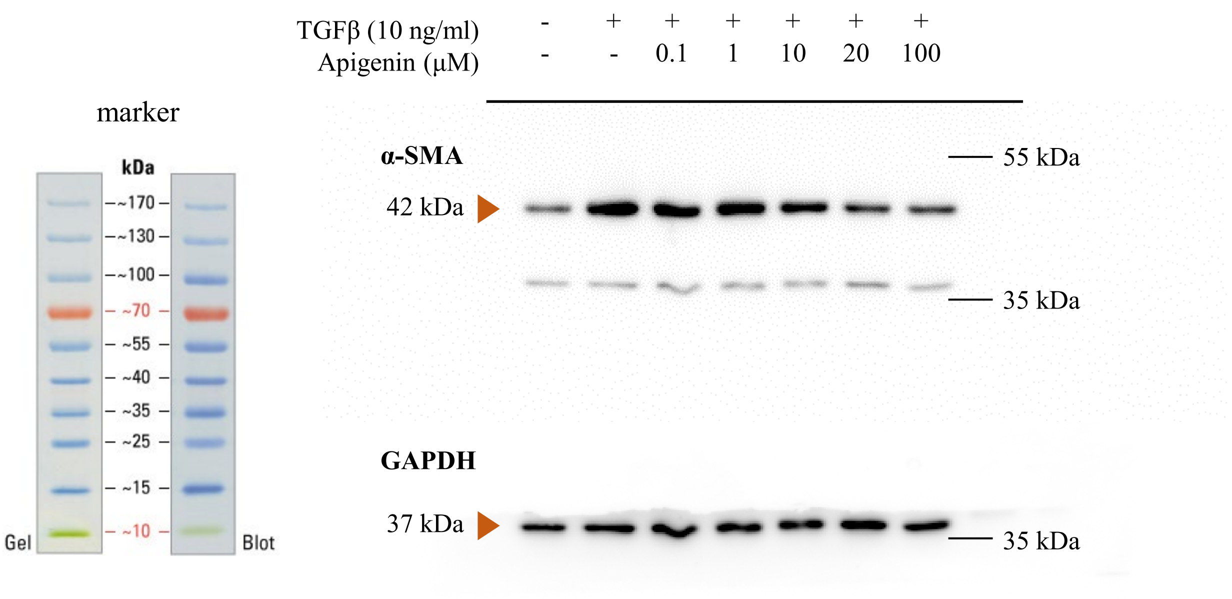

Full unedited blot for Figure 2

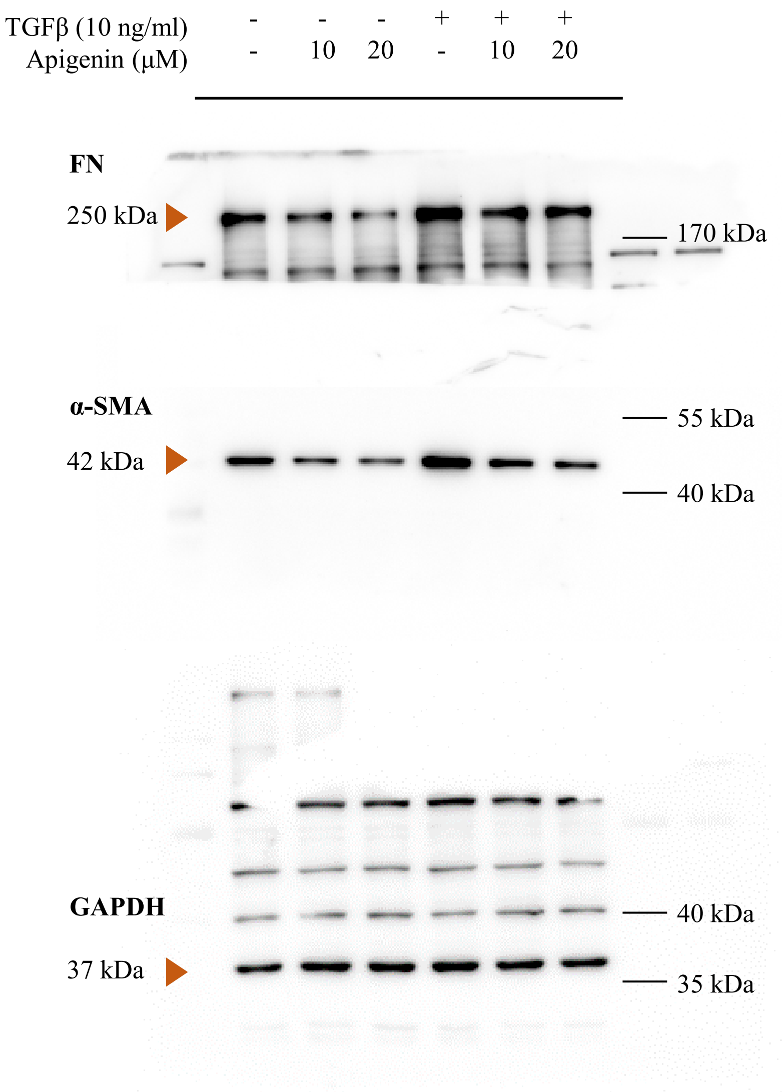

Full unedited blot for Figure 3

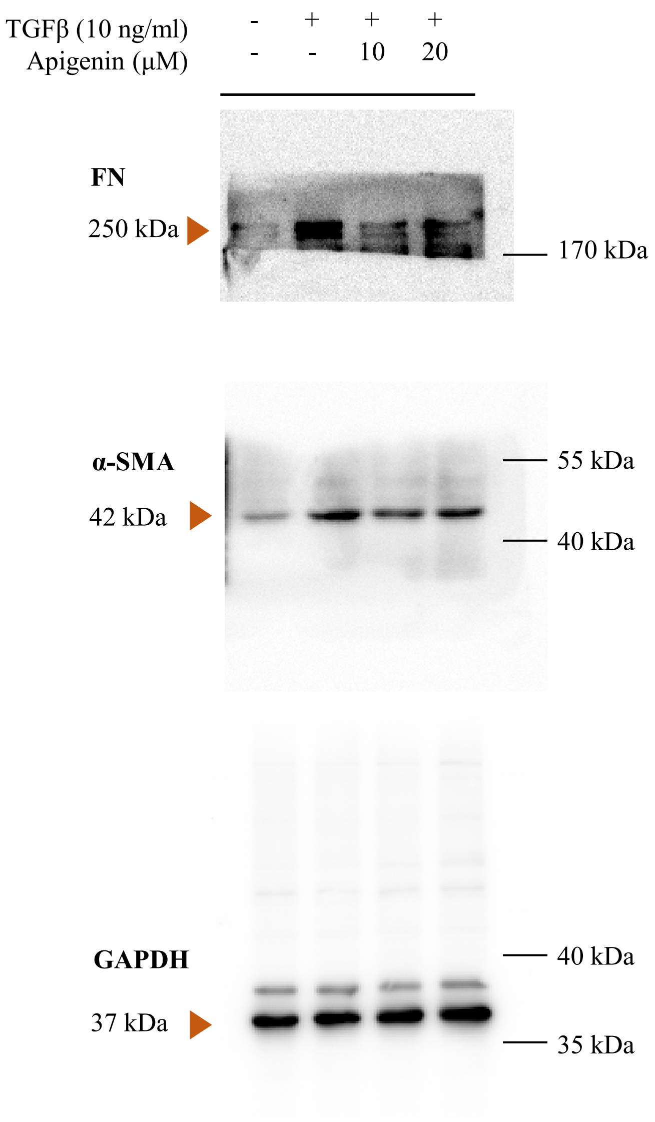

Full unedited blot for Figure 5

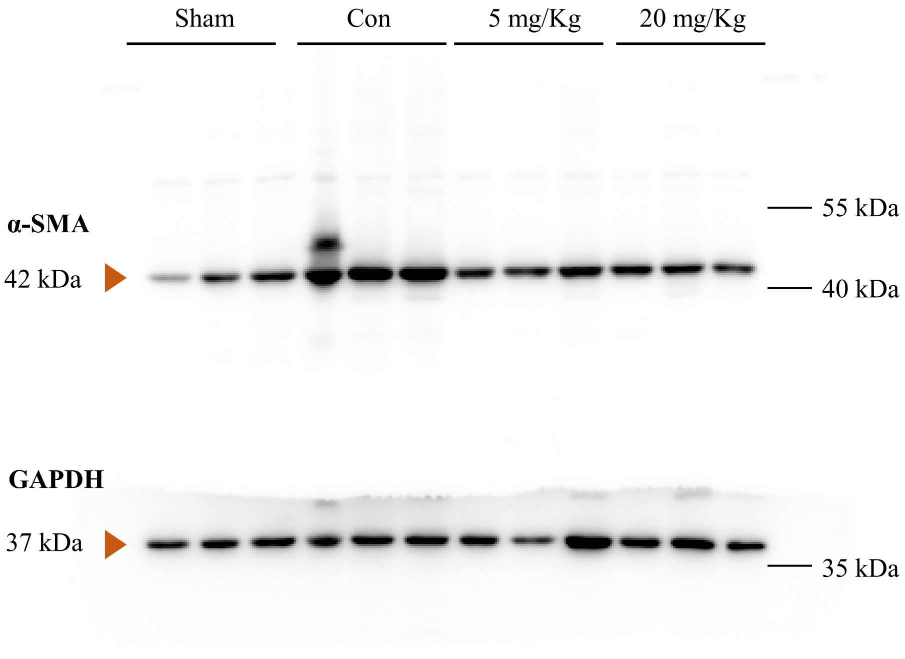

Full unedited blot for Figure 6

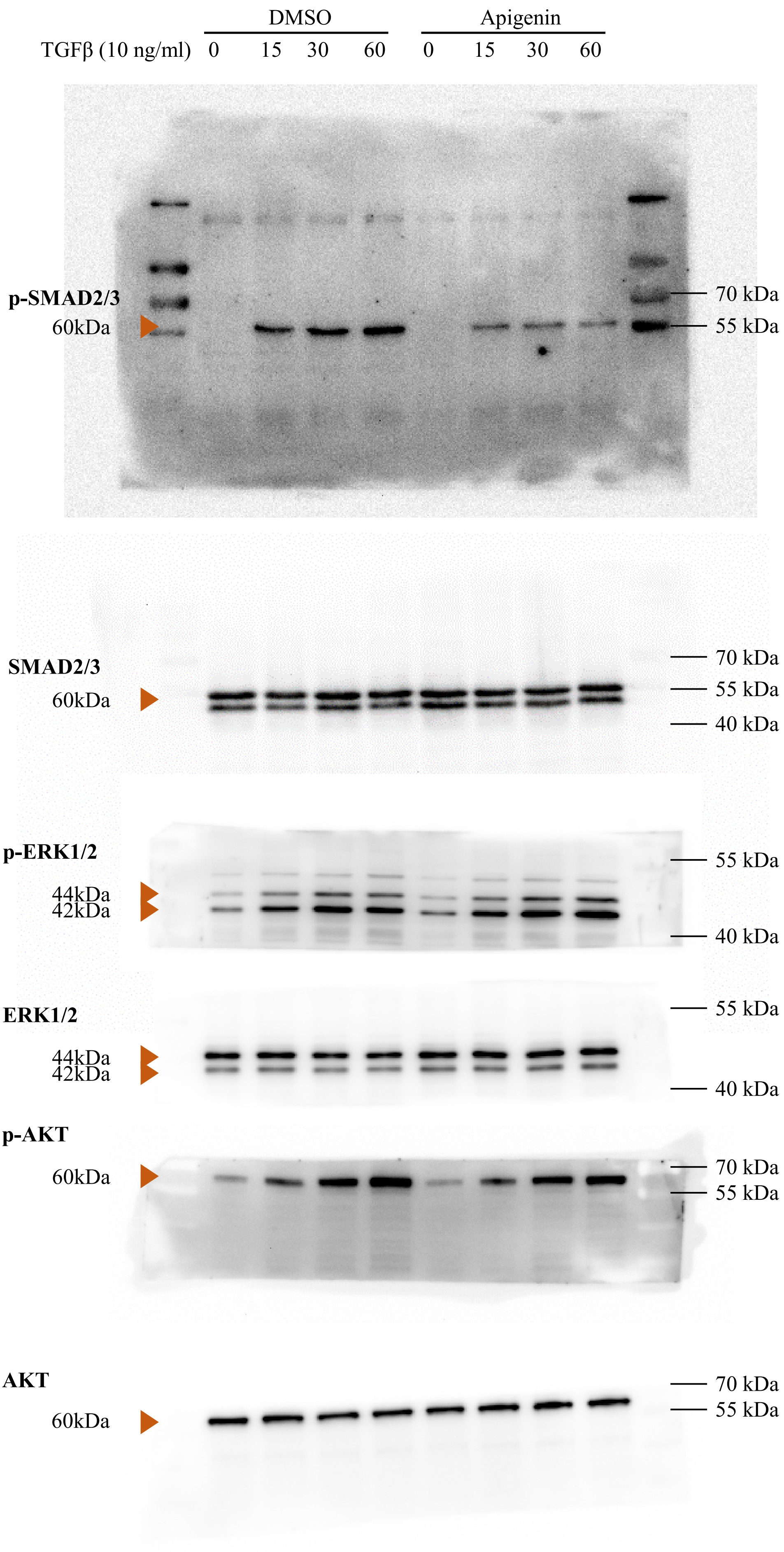

Full unedited blot for Figure 6

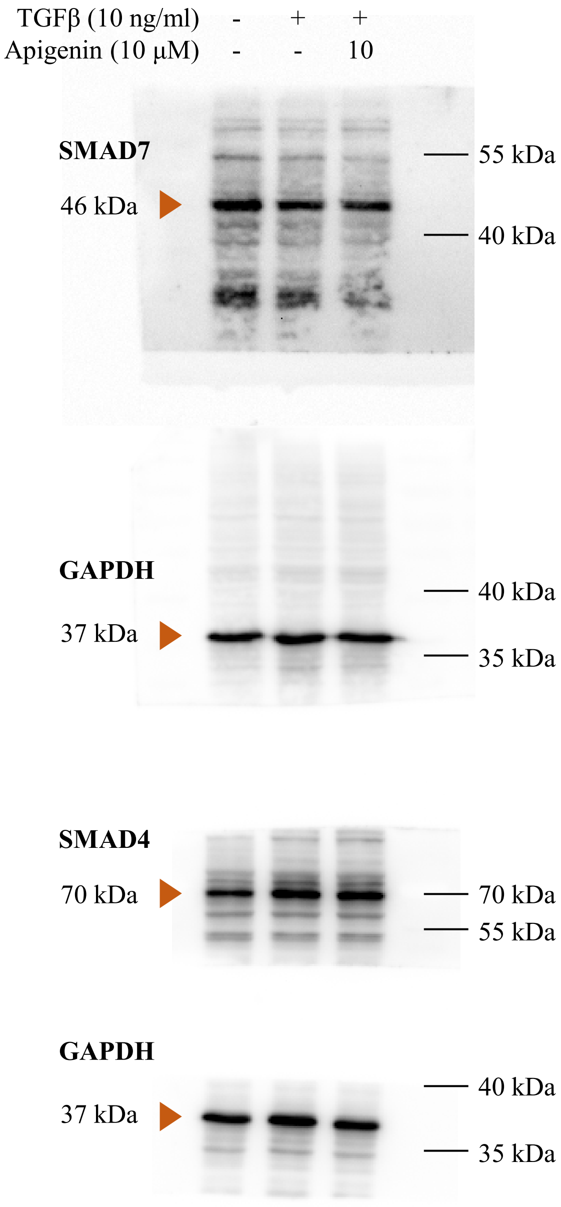

Supplement: Supplementary file 1 — Appendix S1 [file CNS-28-1883-s001.pdf]
